# Evaluation of PLA-Based Composite Films Filled with Cu_2_(OH)_3_NO_3_ Nanoparticles as an Active Material for the Food Industry: Biocidal Properties and Environmental Sustainability

**DOI:** 10.3390/polym16131772

**Published:** 2024-06-23

**Authors:** Xiomara Santos, Gabriela Domínguez, Juana Rodríguez, Javier Pozuelo, Manuel Hernández, Olga Martín, Carmen Fajardo

**Affiliations:** 1 Department of Materials Science and Engineering and Chemical Engineering, Higher Polytechnic School, Carlos III University of Madrid, Avenida Universidad 30, 28911 Leganés, Spain; xsantos@ing.uc3m.es (X.S.);; 2 Department of Biomedicine and Biotechnology, Faculty of Pharmacy, University of Alcalá, Ctra. Madrid-Barcelona km 33.6, 28805 Alcalá de Henares, Spain; gabriela.dominguez@uah.es (G.D.); juana.rodriguez@uah.es (J.R.); manuel.hernandez@uah.es (M.H.)

**Keywords:** compostability, polylactic acid, copper (II) hydroxynitrate, food packaging, bactericidal capacity, food microbiota

## Abstract

The globalization of markets has diversified the food supply, but it has also made the distribution chain more difficult, increasing the risk of microbial contamination. One strategy to obtain safer food and extend its shelf life is to develop active packaging with antimicrobial properties that prevent the growth of pathogenic microorganisms or spoilage in food products. In this context, and in line with the growing social awareness about the environmental impact generated by plastic waste, this work evaluated the effectiveness of polylactic acid (PLA) films loaded with different concentrations of copper (II) hydroxynitrate nanoparticles (CuHS) against the microbiota of fresh foods (chicken, fish and cheese). The results showed that the developed films containing 1, 3 and 5% *w*/*w* of CuHS in the polymeric matrix caused a decrease in the microbial abundance equal to or higher than 3 logarithmic units in all foods tested. Moreover, the mechanical and thermal properties of the formulated composites showed that the added CuHS concentrations did not substantially modify these properties compared to the PLA films. Taking into account the results obtained for antimicrobial activity, Cu (II) migration levels and the cytotoxicity of the films formulated, the PLA composite loaded with 1% CuHS (*w*/*w*) was the most suitable for its potential use as food packaging material. In addition, the biodegradation of this composite film was studied under conditions simulating intensive aerobic composting, demonstrating that almost 100% disintegration after 14 days of testing was achieved. Therefore, the innovative PLA-based films developed represent a promising strategy for the fabrication of packaging and active surfaces to increase food shelf life while maintaining food safety. Moreover, their biodegradable character will contribute to efficient waste management, turning plastic residues into a valuable resource.

## 1. Introduction

Plastics are one of the most important materials in the food packaging industry due to their extensive use. In fact, the current global consumption of plastics exceeds 200 million tons, mainly derived from the petrochemical industry [1]. However, the use of those non-biodegradable polymeric materials causes substantial damage to the environment and a growing problem in the management of plastic waste. In this context, the use of biopolymers such as polylactic acid (PLA) is often the most efficient strategy to face this problem. PLA is a biodegradable and compostable polymer that has been used in the production of packaging materials and is one of the most studied alternatives for the production of renewable materials, substituting products derived from the petrochemical industry [2].

On the other hand, the concern related to food losses and spoilage is reflected with special attention in the 2030 Agenda for Sustainable Development, considering that in 2019 alone, 931 million tons of food were wasted [3,4]. Awareness and action to address this issue is growing daily due to the negative social connotation of unnecessary environmental pollution generated for the production of food that is not consumed. In addition, the continued growth of the world’s population, as well as the more than 820 million people in the world who are suffering from hunger, put additional pressure on the development of alternatives to minimize food losses and waste [3]. Facing this situation would favor the reduction of production costs, improve food security and positively influence the achievement of sustainable development. FAO’s Global Initiative on Food Loss and Waste (Save Food) has conducted studies to identify critical loss points in the food supply chain, where inadequate storage conditions and poor handling practices are one of the main causes [3]. Safer food minimizes potential exposure to illness from microbiological or chemical contamination. In this context, the use of antimicrobial surfaces in food production and handing, as well as the choice of appropriate packaging, are strategies that help to ensure food safety [5]. This is possible because they help to reduce, inhibit or retard the growth of microorganisms and thus increase cost efficiency, as well as extend the shelf life of food products and reduce the environmental problems associated with their production [6,7,8,9].

Therefore, the development of biocidal polymer-particle composites has been widely studied as a promising strategy for obtaining antimicrobial surfaces and packaging [5]. This achievement is likely due to the migration of antibacterial agents, able to inhibit bacterial growth, from the polymeric matrix to the food surfaces [10]. Nanoparticles of silver [11], copper [12], zinc oxide [13], titanium oxide [14] and iron [15] are some of the main antibacterial inorganic fillers used to obtain composites with potential applications in the food industry [5,16]. Particularly, copper, and especially Cu (II) ions, are highly effective species against a wide spectrum of bacteria and fungi [5,9]. Thus, in previous studies carried out by our work group, films based on low-density polyethylene (LDPE) and PLA composites were developed as polymeric matrices filled with copper (II) hydroxynitrate (Cu_2_(OH)_3_NO_3_, CuHS) nanoparticles, to assess their antimicrobial activity [5]. The obtained results proved a high bactericidal capacity against *Listeria monocytogenes* and *Salmonella enterica*, with a reduction of approximately 8 logarithmic units in the films loaded with 0.3% *w*/*w* of the salt [5]. Thus, the characteristics of those innovative developed films could represent a promising biological strategy that fully matches two main requirements of the food packaging industry: to extend the shelf life of food, avoiding microbial contamination, and the replacement of materials derived from the petrochemical industry by more renewable and sustainable ones.

However, despite the antibacterial activity of PLA films with CuHS against pathogenic food-borne microorganisms, the effectiveness of those composites against microbial complex systems needs to be further studied to evaluate their potential application as functional films in the food packaging industry. Moreover, although PLA is considered a biodegradable polymer, it is necessary to evaluate whether the biocidal properties of CuHS nanoparticles could influence the biodegradation process of the novel developed composite. In this context, the aim of this work is to evaluate the feasibility of PLA films filled with CuHS nanoparticles against the microbiota of fresh foods, as well as the environmental sustainability of these new functional systems in terms of biodegradability.

## 2. Materials and Methods

### 2.1. Materials

Copper (II) nitrate 99.999% (Sigma-Aldrich, Madrid, Spain), ethanol 99.5% (Quimipur, Madrid, Spain), acetic acid glacial 99.5% (Panreac, Barcelona, Spain), nitric acid 65% (Scharlau, Barcelona, Spain), dichloromethane 99.9% (Labkem, Barcelona, Spain), MTT dye (3-[4,5-dimethylthiazol-2-yl]-2,5-diphenyltetrazolium bromide) (Sigma Cat. No. M5655, Sigma-Aldrich, Madrid, Spain), Phosphate-Buffered Saline (PBS) (Sigma-Aldrich), liquid nitrogen 99.99% (Linde, Madrid, Spain), Brain and Heart Agar (BHA, Scharlau, Barcelona, Spain), dimethyl sulfoxide 99.9% (DMSO, PanReac AppliChem, Barcelona, Spain), urea 99.5% (Sigma-Aldrich, Spain), saccharose (Scharlau, Spain), filter membranes (Magna, Nylon 47 mm 0.45 µm membrane disk, Fisher Scientific, Waltham, MA, USA) and Doctor Blade (Serial number: 1302004, Neuterk, Madrid, Spain) were used throughout the study. The glass support was obtained from local glassware. Sawdust, rabbit food (based on alfalfa), corn starch and corn oil were obtained from local markets. Mature compost was supplied by the Jardín Botánico of the University of Alcala. Polylactic acid (PLA, Ingeo TM Biopolymer 2003D Nature Works, Blair, NE, USA) was kindly provided by EMSUR (Madrid, Spain). Fresh chicken breast, salmon and cheese were purchased from local supermarkets in Madrid, Spain.

### 2.2. Methods

#### 2.2.1. CuHS Synthesis

The synthesis of CuHS was carried out following a similar procedure reported in previous works [5,17]. Briefly, 20 mL of a 0.1 M copper (II) nitrate solution in ethanol was heated in a microwave (Anton Paar Monowave 400 R, Madrid, Spain) for 6 min at 150 °C. The resultant dispersion was cooled down to room temperature, followed by filtration and subsequent washing three times with distilled water and hot ethanol. Finally, the CuHS was dried in a vacuum oven (Vacuo-Temp, Selecta, Barcelona, Spain) at a pressure of 60 mmHg and 90 °C overnight. 

The CuHS nanoparticles characterization was previously reported in a recent study [5]. The main features determined by FESEM, Raman spectroscopy and X-ray diffraction analyses are shown in Figure S1.

#### 2.2.2. Preparation of PLA/CuHS Composite Films

PLA/CuHS composite films were prepared at room temperature by solution casting with dichloromethane as the solvent, as described in previous works [5,18]. In order to prepare these films, dichloromethane dispersions of CuHS particles (0.3, 1, 3 and 5% *w*/*w* by polymer mass) were prepared; PLA was added up to 10% by mass with respect to the solvent. Adequate particle dispersion was achieved using an ultrasonic treatment (VC 750 Ultrasonic Processor, Thermo Fisher Scientific, Madrid, Spain) for 10 min at 40% of the device power (at intervals of 10 s on, 30 s off). To ensure film thickness homogeneity, a 1000 µm thick Doctor Blade was used on a glass support. The composite films were dried at room temperature for 24 h and then placed in an oven at 40 °C for 5 days to achieve complete solvent removal.

#### 2.2.3. PLA/CuHS Composite Films Characterization

Thermal characterization was carried out using differential scanning calorimetry (DSC) and thermogravimetric analysis (TGA). The DSC analysis was performed with Mettler Toledo DSC equipment (Greifensee, Zürich, Switzerland). The heat treatment for the composite films was performed from 25 to 180 °C at a heating rate of 10 °C/min. Two heating–cooling cycles were performed to remove the thermal history of the material. TGA was carried out on a Perkin Elmer model STA 6000 (Waltham, MA, USA), using a heating cycle from 50 to 600 °C at a rate of 30 °C/min. The tests were performed in an alumina boat under a nitrogen flow rate of 20 mL/min. Both analyses, DSC and TGA, were performed in a nitrogen atmosphere.

The mechanical characterization of the composite films was obtained using tensile tests in isothermal mode at 35 °C to obtain typical stress vs. strain curves. These tests were performed on a TA Instrument Dynamomechanical tester (DMTA), model Q800 (New Castle, DE, USA). Films of 20 × 2 mm (length, width) were tested with striped clamps and a force ramp of 3 N/min up to 18 N to cause the fracture of the specimen. 

Water contact angles of the films were determined using a Drop Shape Analyzer -DSA25 (KRÜSS GmbH, Hamburg, Alemania) equipped with a camera to capture images of the drops. Deionized water was used as solvent to determine the surface wettability of the composite films. Angle measurements were performed in triplicate in three different areas of each sample (right, center and left).

#### 2.2.4. Antibacterial Activity of the Composite Films against Food Microbiota 

For the study of the antibacterial properties of the films, a procedure similar to previous reported by other authors was followed [7,19]. Briefly, native microbiota of chicken breast, salmon and cheese were obtained from 10 g of raw material diluted in 90 mL of Phosphate-Buffered Saline (PBS) using a homogenizer (Laboratory Blender Stomacher 400, Seward, AK, USA). The homogenates, which were made in duplicate from each food, were incubated at 37 °C overnight and then 100-fold diluted. Microbial abundance was determined in each diluted homogenate (control suspension) by plate counting on BHA medium. Hereafter, 100 µL of each diluted suspension was placed in sterile polypropylene Petri dishes and covered with 2 × 2 cm composite films for 24 h at 28 °C. Then, 10 µL of the bacterial suspension were tenfold diluted and cultured on BHA for 24 h at 37 °C. The bacterial colonies were then counted, and the CFU/mL were calculated. The results were expressed as a mean logarithmic (log) reduction by comparison with the PLA films. The log reduction was calculated by subtracting the logarithm in base 10 of the CFU/mL determined on the bacterial suspension covered with the PLA films, and the logarithm in base 10 of the CFU/mL obtained on the bacterial suspension covered the PLA/CuHS composite films. 

#### 2.2.5. Migration Assay and Swelling

The specific migration test for copper and swelling of the different composite films was performed with liquid food simulant media—food simulant C for aqueous and alcoholic foods (10% *v/v* ethanol) and food simulant B for acidic foods (3% *v/v* acetic acid). The specific migration was determined according to Regulation (EU) No. 10/2011 (EC, 2011) and as described in previous works [5,20,21]. The assay consisted of the total immersion of the films with a surface of approximately 4 cm^2^ in the food simulants for 10 days at 40 °C. Subsequently, the films were removed, and 1 mL of the media was diluted with Milli-Q water in the presence of 0.5 mL nitric acid (5% *v/v*) to complete 10 mL. This dilution was used for the Cu quantification using inductively coupled plasma-optical emission spectroscopy (Varian—Agilent 720 ICP-OES, Dörentrup, Germany). The regulation used to determine copper migration establishes a specific migration limit of 5 mg/kg for samples of 6 dm^2^. The necessary corrections were made taking into account that our films had a size of 0.04 dm^2^.

In addition, an absorption test was performed to determine the swelling properties of the samples in the two food simulants above described. The films were weighed on day 0 (before immersion in the ethanol and acetic acid solutions). After 10 days of contact with both media, the films were recovered, dried with filter paper, and weighed again. Swelling was determined as the percent of water mass absorbed with respect to the initial mass weighed of each sample.

#### 2.2.6. Cytotoxicity Assay 

Cytotoxicity of the composite films was evaluated at 24 h using the Cell Proliferation Kit I (MTT): Colorimetric assay for the non-radioactive quantification of cell proliferation and viability, using HeLa cells seeded in multiwell plates [5]. To determine cell viability, MTT dye was used, which was dissolved to obtain a 5 mg/mL solution in PBS. This solution was filtered through a 0.2 µm filter and stored at 2–8 °C. Routinely, MTT stock solution (5 mg/mL) was added to each culture to be tested to equal one-tenth of the original culture volume and incubated for 3 to 4 h. At the end of the incubation period, the medium was removed to work with adherent cells. The converted dye (from MTT to purple formazan via the interaction with the live cells’ dehydrogenase enzymes) was solubilized in dimethyl sulfoxide (DMSO). The absorbance of the converted dye was measured at a wavelength of 570 nm (FluoStar Omega, BMG LabTech, Ortenberg, Germany).

#### 2.2.7. Disintegration Test under Aerobic Composting Conditions

To evaluate the biodegradability of the developed composite, a disintegration test under aerobic composting conditions was conducted according to the European standard UNE-EN ISO 20200:2004 [22]. In brief, a synthetic solid residue was prepared and inoculated with mature compost (Table S1). Then, six bioreactors were set up as following: 2 containing no plastic material, used as control, 2 with PLA samples and 2 with PLA/CuHS 1% *w*/*w*. Both PLA and PLA/CuHS films were prepared with dimensions of 2 × 2 cm, introduced in bags with a mesh size < 1.5 mm, and added to the bioreactors at a concentration of 0.5% *w*/*w*. The bioreactors were incubated at 58 ± 2 °C for 45 days under controlled conditions of aeration and relative humidity. The synthetic solid residue and plastic samples were collected on days 2, 4, 7, 14, 21, 30 and 45 of the test. At each sampling time, the pH was measured in the solid residue and the disintegration degree of plastics was determined by reduction in mass. Evolution of the thermal and mechanical properties of the plastic residues was studied as described in Section 2.2.3, and their surface characteristics were determined by FESEM (Hitachi SU-70; Chiyoda, Tokyo, Japan) using secondary electron detection and an incident electron beam voltage of 5 kV.

At the beginning and the end of the test, the solid residue was characterized in terms of total organic carbon content, total nitrogen (LECO CHNS-932 Analyser¸ LECO Instruments S.L, Tres Cantos, Spain), dry mass and volatile solids. The dry mass was obtained by taking a known volume of test material which was dried at 105 °C to constant mass and the amount of volatile solids was determined by subtracting the residue obtained from a known volume of compost from the total dry solids content of the same sample after incineration at 550 °C.

### 2.3. Statistical Analysis

The obtained results were expressed as the mean ± standard deviation. An analysis of variance was carried out using the Tukey test to determine significant differences at a 5% significance level (*p* < 0.05) using the statistical software JMP Pro 15 (SAS Institute, Cary, NC, USA).

## 3. Results and Discussion

### 3.1. PLA/CuHS Composite Films Characterization

As above mentioned, the PLA film with CuHS 0.3% (*w*/*w*) was fully characterized in a previous study carried out by our research group [5]. Thus, the thermal and mechanical characteristics of the PLA/CuHS 1, 3, 5% (*w*/*w*) films were assessed in this work. 

Related to the study of the thermal properties of films, the results show that the maximum decomposition rate temperature (Td on peak) is very similar for all the films studied (Figure S2a and Table 1). However, the decomposition characteristics of the polymer (PLA) are clearly affected with the addition of salt. This effect is attributed to the presence of the nanoparticles, which due to their high specific surface area can cause local heating capable of accelerating the polymer decomposition process [5,18,23]. For example, it is observed how the decomposition process starts to occur (Td_onset_) at lower temperatures as the percentage of CuHS increases, leading to a two-step decomposition process. The percentage of mass loss of the first decomposition step increases as the salt content increases, related to the ability of CuHS to catalyze the decomposition of the PLA polymeric matrix. This phenomenon was observed in previous studies carried out by our work group on PLA films with 0.3 and 0.5% (*w*/*w*) of CuHS [5]. 

The glass transition temperature (Tg) of PLA and the obtained composites films are quite similar and do not appear to trend with the amount of salt added (Figure S2b and Table 1). Comparing the Tg values of the PLA films obtained in this work by solution casting and the commercial PLA (which was not in contact with the solvent), it can be observed that both are very similar (Figure S3). This finding corroborates that an adequate removal of the solvent was achieved, since no plasticizing effect (decrease in Tg) is observed due to the presence of the small solvent molecules. Cold crystallization and melting for both PLA and composites start at very similar temperatures (Tc and Tm_onset_). However, there seems to be a slight increase in the maximum rate temperatures of both cold crystallization and melting (Tc and Tm on peak) with the presence of CuHS in the PLA matrix. In addition, the curves shift towards higher heat flow values in the presence of the salt. The aforementioned results are similar to those obtained in the thermal characterization of PLA composites reinforced with hematite (α-Fe_2_O_3_) [24], due to the addition of the inorganic filler disfavoring the crystallization process of the PLA matrix. In our case, this fact can be even ascertained with the decrease of both cold crystallization enthalpy and melting enthalpy (ΔHc and ΔHm) in the films containing 1, 3 and 5% (*w*/*w*) of CuHS.

Table 2 summarizes the mechanical properties of PLA and composites films obtained from the analysis of stress vs. strain curves. An increase in the maximum stress and Young’s modulus (or elasticity) is observed, which translates into an increase in the brittleness of the composite films with respect to PLA. This behavior is characteristic of systems where, due to the weak interaction between the polymer and the filler, a stress concentration occurs since the particles act as voids in the polymer matrix [5,18]. Nevertheless, the obtained results of Young’s modulus are in agreement with those reported in previous works studying the mechanical properties of PLA films with different nanofillers, with E values ranging from 1.6–10.8 GPa [5,18,25,26,27]. Moreover, the values of maximum stress and strain, as well as the Young’s modulus of our new materials are within the range reported by other authors who evaluated the suitability of PLA and PLA composites developed for food packaging applications [28,29,30].

**Figure 1:**
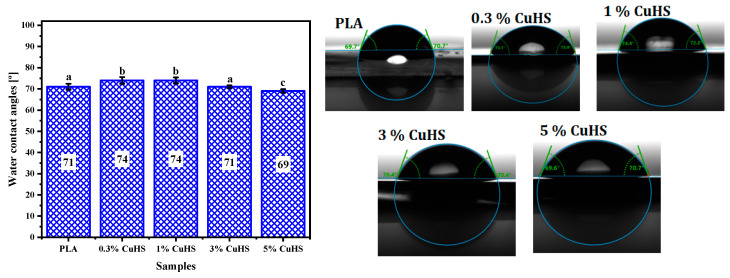
Water contact angle (WCA) of PLA and PLA/CuHS composite films. Data represent the mean values and standard deviation (*n* = 9). Identical letters mean no statistical significance, while different letters mean statistical significance (*p* < 0.05).

Figure 1 shows the contact angles of the films determined using water as solvent (WCA). The absorption of water by the surface of a material is related to the wettability and, therefore, to its hydrophilic/hydrophobic character [31]. A better wettability/hydrophilicity of the material implies a larger contact area between the droplet and the surface of the material. Therefore, a lower contact angle implies a higher wettability [31,32]. The results obtained in this study do not show a clear pattern in the WCA values for the CuHS nanoparticle-added films compared to the PLA. Even though the WCA values for the samples containing 0.3, 1 and 5% CuHS (*w*/*w*) differ statistically from the value obtained for PLA, the results are very similar and are in agreement with those reported by other authors [32]. The contact angle depends on two fundamental factors: the morphology and chemical characteristics of the surface; in this context, according to Valerini et al., the high contact angle values for PLA films can be related to the low degree of polarity of its surface [32].

### 3.2. Antibacterial Capacity of the Composite Films against Food Microbiota 

Microbiological contamination is one of the main causes of food loss and waste [7] and therefore the development of new systems for the control of microorganisms in fresh products has gained increasing attention. For instance, chicken is a natural reservoir for bacteria such as *Salmonella* and *Campylobacter* [8,33,34], while seafood products are highly susceptible to bacterial contamination due to their high water and free amino acid content, leading to spoilage of fish flesh, especially when improperly processed or stored [35]. Similarly, pathogens such as *L. monocytogenes* and *Escherichia coli* have been detected in dairy products, as well as sheep and goat cheeses [36]. Therefore, ensuring food safety and extending the shelf life of these products is a challenge that involves the development of strategies to reduce their microbial content. 

According to our previous results on the biocidal properties of the CuHS nanoparticles, in this study, PLA composite films with CuHS nanofiller in the range of 0.3–5% *w*/*w* were prepared. To evaluate the antibacterial capacity of the new films, the microbiota of fresh food, chicken breast, cheese and fish (salmon) was used to simulate more complex microbiological systems, similar to the environments where films would be placed for food packaging. 

**Figure 2:**
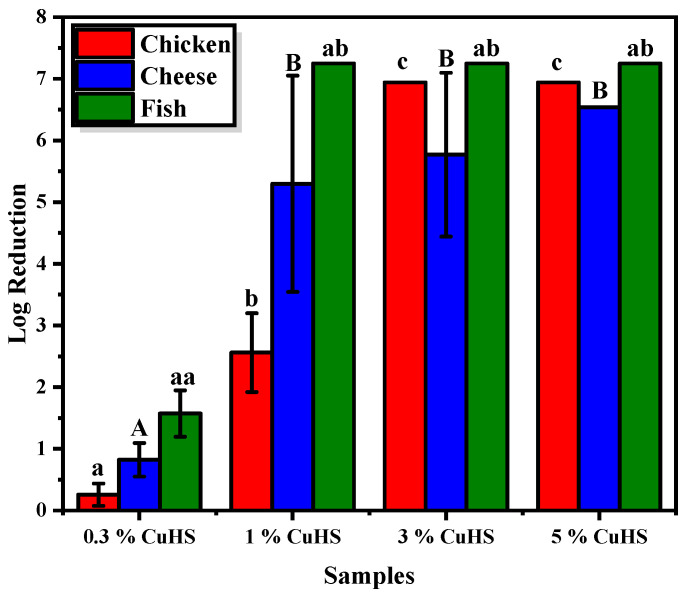
Logarithm reduction of CFU/mL obtained on the chicken breast, cheese and fish bacterial suspensions covered by PLA/CuHS films. Data represent the mean values and standard deviation (*n* = 4). Identical letters mean no statistical significance, while different letters mean statistical significance (*p* < 0.05).

Table S2 shows the microbial abundance (log CFU/mL) determined in the initial control suspensions (diluted homogenates), and in the bacterial suspensions after 24 h of contact with each of the tested plastic samples. The measured concentration of microorganisms in the suspensions covered with the PLA films were 8.69 × 10^6^, 3.44 × 10^6^ and 1.78 × 10^7^ CFU/mL for the chicken, cheese and fish foods, respectively. These results highlight that the PLA film does not have any biocidal properties; in fact, it can be observed how the number of microorganisms increases with respect to the control sample. This finding is likely due to the presence of residual organic matter in the suspension, which causes an increase in the microorganism’s population. Thus, taking into account this result, the microbial counts determined after contact with the PLA films were chosen as the reference, for comparative purposes, to determine the reduction of the microbial community caused by the PLA/CuHS composite films (Figure 2).

The results obtained clearly showed that the biocidal properties of the composite were related to the concentration of CuHS within the polymeric matrix. The film PLA/CuHS 0.3% (*w*/*w*), the optimal CuHS concentration with proven activity against pure cultures of food-borne microorganisms [5], shows scarce antibacterial activity against the microbiota of the fresh foods tested. Only a log reduction of approximately 1.57 is observed for the fish microbial extract. These results indicate that the antimicrobial activity of the developed composite against food microbiota was less remarkable than that found in the in vitro tests with pure culture microorganisms, according to the results obtained by Requena et al. related to the antibacterial properties of poly(3-hydroxybutyrate-co-3-hydroxyvalerate) (PHBV) films added of eugenol and carvacrol [37]. This result could be due to the presence of the organic matter contained in the bacterial suspensions in contact with the films, which could immobilize copper ions, reducing their bioavailability and therefore their potential antimicrobial activity. Thus, the use of higher concentrations of the biocidal agent to obtain active materials against food microbiota is needed.

In the PLA/CuHS 1% (*w*/*w*) films, a reduction of approximately 3 logarithmic units is observed in the chicken microbiota, reaching values of 5.3 and 7.3 for the cheese and fish microbial extracts, respectively. In the case of PLA films with 3 and 5% CuHS (*w*/*w*), as expected due to the higher salt concentration in the matrix, an increase in the antimicrobial capacity against the chicken microbiota is observed, reaching in both conditions the maximum possible log reduction according to experimental design of the test (a reduction of 6.9 log units). However, for the cheese and fish microbiota no significant differences among the assessed CuHS doses (1, 3 y 5% *w*/*w*) were recorded, leading in all cases to a log reduction higher than five units. Thus, for these food matrices the 1% CuHS (*w*/*w*) concentration is high enough to obtain active materials, particularly for fish, enabling to achieve the maximum expected log reduction according to the design of our experiment (7.3). Overall, the obtained results point out that the introduction of the CuHS nanoparticles in the PLA matrix, either at a dose of 1, 3 or 5% (*w*/*w*), leads to a decrease in the initial food microbial population greater than three logarithm units, demonstrating its biocidal activity against fresh-food microbiota [38], and supporting their potential use as an alternative for obtaining active plastic packaging.

### 3.3. Cu (II) Migration, Swelling and Cytotoxicity of the Composite Films

Besides, an extremely important aspect hat is closely related to food quality is food safety. A food product must be guaranteed to be safe for the consumer, and thus special attention must be paid to any possible risk arising from the use of active agents from metallic sources [9]. Cu (II) is known to be effective against microbial cells due to its possible interaction with nucleic acids and cell membrane components causing cell death [9,39]. Thus, the observed biocidal activity of CuHS could be expected to be primarily due to the migration of Cu (II) ions; since the cell wall of bacteria is negatively charged, positively charged nanoparticles or ions may adhere better to the bacterial cell walls and inactivate bacteria more efficiently [40]. Moreover, migrated Cu (II) ions could induce cellular oxidative stress because, in its oxidized state, Cu (II) participates in reactions in which free hydroxyl radicals are produced, in addition to superoxide anion and hydrogen peroxide [5,20,41]. These reactive oxygen species (ROS) attack the membranes through a lipid peroxidation process, causing the destabilization of their structure and affecting their cellular functions [42].

Then, it is essential to study the migration of Cu (II) ions to evaluate the potential cytotoxicity of the composite films developed in order to demonstrate the safety of the proposed system for their potential application in the food packaging industry. 

**Figure 3:**
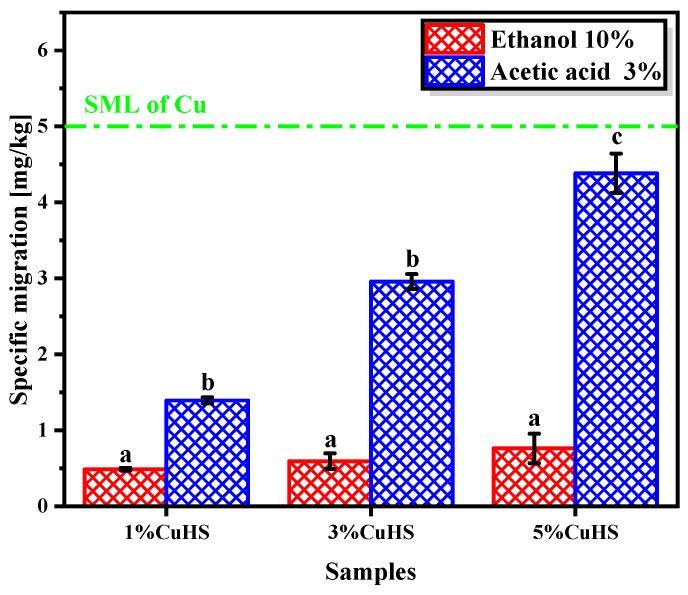
Copper-specific migration in two food simulants (ethanol 10% and acetic acid 3% v/v). Data refer to the standardized specific migration limit (SML), set at 5 mg/kg. Data represent the mean values and standard deviation (*n* = 5). Identical letters mean no statistical significance, while different letters mean statistical significance (*p* < 0.05).

Figure 3 shows the results corresponding to the study of migration Cu (II) from the developed films. As expected, the increase of the amount of CuHS in the polymeric matrix increases the migration of Cu (II) (Figure 2); moreover, the obtained results show that the highest copper migration is observed when acetic acid (3% *v*/*v*) is used as food simulant. This result agrees with similar studies found in the literature, where the migration of metal ions is higher at acidic pH [5,43]. However, even in the composite formulated with 5% CuHS (*w*/*w*), a specific migration of 4.38 mg/kg (in acetic acid) is obtained, which is below the allowed specific migration limit (SML) which is 5 mg/kg.

In addition, the swelling properties of the developed films in the food simulants above stated were assessed to mimic the possible absorption of liquid under real conditions. The results show that the addition of CuHS nanoparticles to PLA did not affect its absorption capacity in the tested media (ethanol 10% *v/v* and acetic acid 3% *v/v*) and no statistically significant differences among the samples were recorded. In fact, the absorption was almost null for all the samples, which demonstrates the hydrophobicity of the developed films. This characteristic is essential for materials with potential applications in the food packaging industry, since it limits the diffusion of water molecules in the polymeric matrix [31].

**Figure 4:**
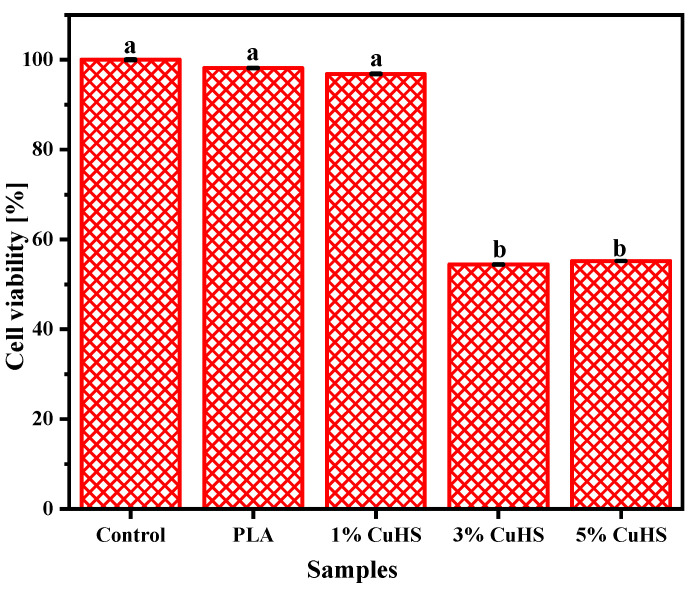
Viability of HeLa-like cells after exposure to the developed composite films. Data represent the mean values and standard deviation (*n* = 3). Identical letters mean no statistical significance, while different letters mean statistical significance (*p* < 0.05).

Figure 4 shows the results corresponding to the analysis of the cytotoxicity of the films against the HeLa cell line, whose cells are related to cervical uterine cancer. This test is considered essential because, although copper (II) migration was below the permitted limit in all cases, the salt contains other ions that could have a negative effect on the safety of food products that are in contact with the formulated active material. In the case of PLA and PLA films with 1% CuHS (*w*/*w*), a cell viability of over 96% is obtained, which highlights that these systems are safe from a cytotoxic point of view. However, the films with 3 and 5% CuHS (*w*/*w*) cause a loss in cell viability of 46 and 45%, respectively, which means that the number of live or metabolically active cells is below 70%, and therefore they are considered as cytotoxic materials [5,18,44].

### 3.4. Biodegradation Assessment under Composting Conditions

Considering the previously obtained results, it has been demonstrated that PLA/CuHS 1% (*w*/*w*) exhibited an effective biocidal activity against the microbiota of the tested foods. Furthermore, taking into consideration that the addition of this amount of salt is safe in terms of both copper ion migration and cytotoxicity, it can be concluded that this would be the optimal system to be used as a safe biocidal material for being in contact with food. Thus, for further assays, to test the compostability of the new formulated films, the PLA composite containing CuHS 1% (*w*/*w*) was chosen to determine the influence of the salt on the biodegradation process of the polymeric matrix. 

#### 3.4.1. Disintegration Process 

**Figure 5:**
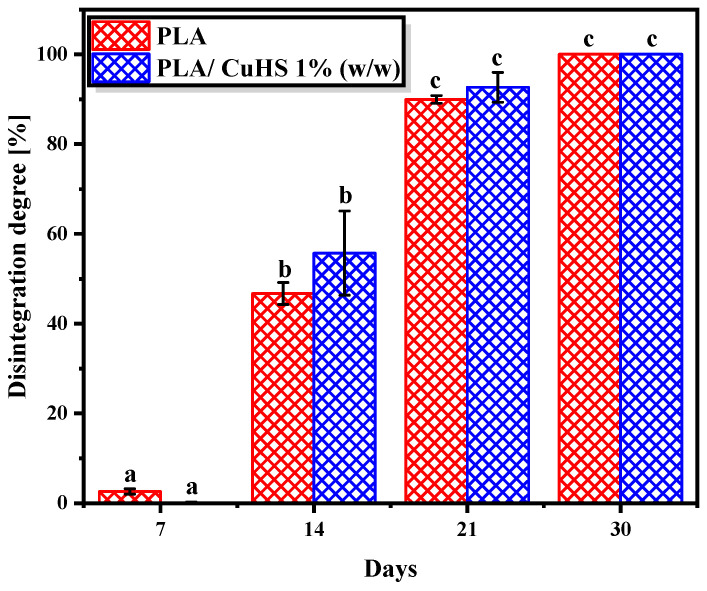
Percentage of disintegration of PLA and PLA/CuHS 1% (*w*/*w*) samples at different sampling times. Data represent the mean values and standard deviation (*n* = 4). Identical letters mean no statistical significance, while different letters mean statistical significance (*p* < 0.05).

**Figure 6:**
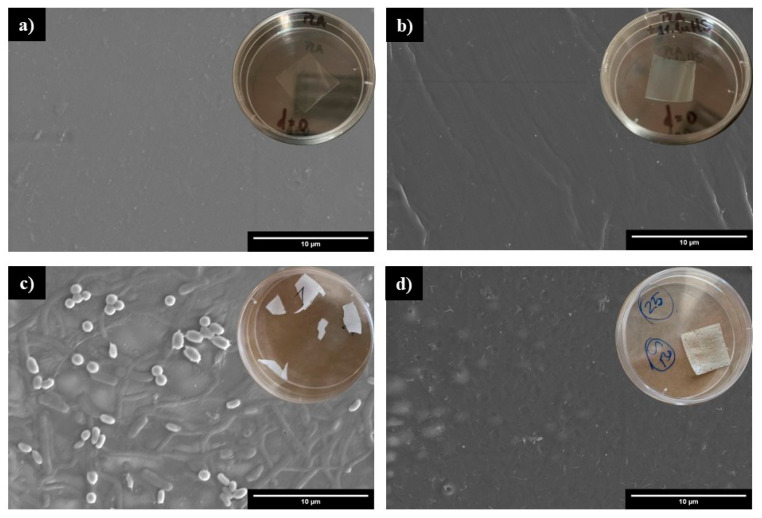

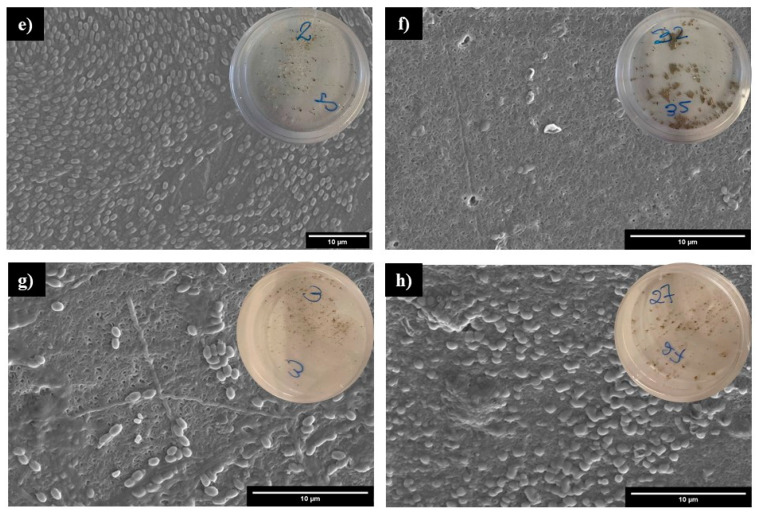
Macroscopic and microscopic appearance (determined by FESEM) of plastic residues at different times of the biodegradation test. Day 0 (**a**) PLA; (**b**) PLA/CuHS 1% (*w*/*w*). Day 7 (**c**) PLA; (**d**) PLA/CuHS 1% (*w*/*w*). Day 14 (**e**) PLA; (**f**) PLA/CuHS 1% (*w*/*w*). Day 21 (**g**) PLA; (**h**) PLA/CuHS 1% (*w*/*w*).

The results obtained for the disintegration degree of the composite films, determined by mass loss along the incubation period, are shown in Figure 5. By day 7 of testing, the PLA samples were extremely brittle, and their degradation appeared to be more noticeable than that observed in the films loaded with 1% *w*/*w* CuHS. By day 14 of testing, both plastic samples showed significant disintegration progress, with a loss in the mass weight of 47 and 56% for PLA and PLA/CuHS 1% (*w*/*w*) samples, respectively (Figure 5). However, considering that all the plastic residues collected at this day were equal or smaller than 2 mm (Figure 6e,f), which according to the standard are considered degraded material, after 14 days 100% of degradation was recorded for all the assessed plastic samples. These results are in agreement with other works reported in the literature. For instance, the degradation of PLA and PLA nanocomposites reinforced with cellulose nanocrystals tested in compost were reported to be greater than 90% after 14 days of assay [45].

On day 21 of the test, a scarce fraction of the initial plastic samples was collected from the bioreactors (with a size ≥ 1.5, ≤2 mm) due to the advanced degradation presented by PLA and PLA/CuHS samples as above mentioned. On subsequent days (30 days of incubation), it was not possible to obtain samples because the fragments of plastic formed were small enough to pass through the mesh of the bags where they were placed in order to track the degradation process (fragments < 1.5 mm). Moreover, no statistically significant differences between the two films studied were found along the composting assay, suggesting that the biocidal properties of the inorganic filler do not affect the biodegradable characteristics of the PLA matrix.

#### 3.4.2. Characterization of Plastic Residue 

The macroscopic and microscopic appearance of the plastic samples was analyzed at 0, 7, 14 and 21 days of the biodegradation test (Figure 6). A change in coloration of the samples was observed even in the first 7 days of the process. Initially, the films were transparent and then became fundamentally opaque/white, likely due to the fact that, during the hydrolytic degradation of PLA, chain scission reactions are favored within the amorphous regions, which results in an increase in the crystallinity of the polymer [46]. Besides, during the hydrolytic process, the water absorption and/or the formation of low molecular weight products lead to a change of the refraction index of the materials [47].

Figure 6 also shows the images obtained by FESEM, where we observed how the surface of the PLA films is colonized by microorganisms, even at day 7 of testing (Figure 6c). However, for PLA/CuHS films, the presence of microorganisms on its surface at days 7 and 14 is limited, suggesting that the antimicrobial capacity of the salt could hinder microbial colonization (Figure 6d,f). 

PLA degradation can be considered a three-step process, where an initial biodeterioration of the polymer occurs due to the microbial colonization of its surface. Subsequently, physic–chemical and/or microbial degradation (by the release of hydrolytic enzymes) takes place, leading to a fragmentation of the polymer. In a third phase, the microbial community mineralizes the released compounds from cleavage of the ester bonds [48]. 

Therefore, considering that the degree of disintegration of the samples along the composting process (determined as mass loss) are not statistically different, the delay in the microbial colonization detected in PLA/CuHS films, might not be a pre requirement for PLA degradation. On the other hand, microbial degradation mechanisms are favored after the high molecular weight PLA chains are fragmented by hydrolysis [46], which could explain that, on day 21 of testing, even the plastic residues from the CuHS films are colonized by microorganisms. Thus, the CuHS nanofiller does not affect the degradation process of the PLA polymeric matrix, which is likely mainly due to the enzymatic and/or physical-chemical hydrolysis of ester bonds under the appropriate humidity conditions.

Additionally, the pH was monitored through the composting process (Figure S4). The values measured in all samples remained above 7.5 during the test, indicating a good decomposition and correct aeration of the mixture. The highest values were recorded between days 7–21 of incubation, according to the higher disintegration rate and likely to the higher microbial activity, exhibited in this period. After 21 days, all the samples tend to neutrality, particularly the PLA and PLA/CuHS added bioreactors, reaching values below 8, which are usual final pH values in a composting process. 

In order to further study the degradation of the films, the plastic residues were thermally and mechanically characterized at 0, 2, 4 and 7 days of the composting test. Table 3 shows the different parameters obtained from the thermogravimetric analysis and differential scanning calorimetry of the first heating process. The initial PLA matrix was fundamentally amorphous, with a noticeable glass transition temperature (Tg) and a small melting peak (Tm). In addition, when we analyze the Tg values, a trend to decrease is observed in the first 7 days of incubation, compared to the Tg values of the films before composting (day 0); this can be explained due to an increase in the mobility of the polymer molecules resulting from the hydrolysis process and the plasticizing effect of the water and the smaller fragments [2,49,50]. For instance, in the PLA sample after 7 days of incubation, the signal corresponding to the Tg disappears suggesting the entire degradation of the amorphous part. This finding is supported by the previously mentioned results on the color changes observed in the films, pointing, as above explained, that the degradation of PLA-based materials begins in the amorphous region of the polymer [46]. This increase in crystallization during the composting process is also evident when analyzing the enthalpy of fusion, which increases substantially. Furthermore, Figure S5 shows how in PLA and PLA/CuHS samples at days 4 and 7 of the composting process the melting peak is bimodal, which implies the formation of smaller and imperfect crystals [2].

On the other hand, a study of the mechanical properties of the films was carried out on days 2 and 4 of the composting test, since it was impossible to do so on later days due to the high brittleness of the samples. Table 4 summarizes the results obtained from the analysis of the stress vs. strain curves where an accentuated deterioration of the determined magnitudes in the PLA samples is observed. In the case of PLA/CuHS films although there is a clear tendency to decrease, there are no statistically significant differences among the parameters determined (Young’s modulus, maximum stress and strain) at days 2 and 4 of incubation.

#### 3.4.3. Compost Characterization

The resulting compost from each bioreactor was passed through 10, 5 and 2 mm sieves to determine if there were any plastic residues larger than these dimensions. In none of the cases was it possible to isolate plastics with dimensions larger than those of the sieves used. Finally, the composts resulting from the biodegradation process of the films were physic-chemically characterized. Table 5 shows the results obtained for the parameters determined. There were no statistically significant differences between samples in terms of final dry mass or volatile solids content. Moreover, in all samples the decrease in the volatile solids content at the end of the test compared to the initial content (R value), is higher than 30%, which confirms the correct performance of the composting test. Besides, the carbon to nitrogen (C/N) ratio is a significant parameter in composting because microorganisms need a good balance of carbon and nitrogen (ranging from 25 to 35) in order to remain active [51]. The C/N ratios of the final residues obtained in this study are in all samples within this range, pointing out their feasibility as soil amendment materials.

## 4. Conclusions

In this study, we demonstrated the biocidal properties of PLA-based composite film filled with CuHS nanoparticles (1% *w*/*w*) against fresh food microbiota, and its safety in terms of copper migration and cytotoxicity. Thus, the obtained results indicate the suitability of the new formulated composite for its use as an innovative material to increase the shelf life of food by preventing undesirable growth of microorganisms. Furthermore, the thermal and mechanical properties of the analyzed composite films support its potential application for the fabrication of active packaging. On the other hand, we have proved that the developed functional film is biodegradable under composting conditions, and thus the biocidal properties of the salt do not alter the normal composting process of the polymeric matrix. 

Therefore, the technological approach developed in this study may provide a promising strategy to produce functional food packaging materials, contributing to the replacement of fossil fuel-based plastics by a more sustainable and environmentally friendly alternative, as well as to the revalorization of the generated plastic wastes producing suitable soil improvers.

## Figures and Tables

**Table 1:** Parameters related to the study of the thermal properties of films. DSC parameters from the second heating process.

	Samples	PLA	PLA/CuHS 0.3% (*w*/*w*)	PLA/CuHS 1% (*w*/*w*)	PLA/CuHS 3% (*w*/*w*)	PLA/CuHS 5% (*w*/*w*)
Parameters						
Tg [°C]		57	57	59	60	58
Tc_onset_ [°C]		106	106	110	108	110
Tc_on peak_ [°C]		118	120	126	126	127
Tm_onset_ [°C]		142	143	147	146	146
Tm_on peak_ [°C]		148	149	151	152	152
ΔHc [J/g]		−16	−17	−7	−4	−4
ΔHm [J/g]		16	17	6	3	4
Td_onset_ [°C]		355	349	297	295	295
Td_on peak_ [°C]		373	379	374	377	377
Ref.		This study	[5]	This study		

**Table 2:** Main results obtained from the mechanical analysis of the composite films tested. Data represent the mean values and standard deviation (*n* = 5). Identical letters within a column mean no statistical significance, while different letters mean statistical significance (*p* < 0.05).

Samples	σ_max_ [MPa]	ε_max_ [%]	E [GPa]
PLA	40 ± 10 ^a^	15 ± 6 ^A^	2.5 ± 0.4 ^aa^
PLA/CuHS 1% (*w*/*w*)	70 ± 7 ^b^	12 ± 5 ^A^	3.5 ± 0.5 ^ab^
PLA/CuHS 3% (*w*/*w*)	65 ± 8 ^b^	16 ± 6 ^A^	3.4 ± 0.4 ^ab^
PLA/CuHS 5% (*w*/*w*)	65 ± 6 ^b^	15 ± 3 ^A^	3.5 ± 0.4 ^ab^

**Table 3:** Parameters related to the study of the thermal properties of the plastic residues from the composting test. DSC parameters obtained from first heating process.

	Parameters	Tg [°C]	Tm_onset_ [°C]	Tm_on peak_ [°C]	ΔHm [J/g]	Td_on peak_ [°C]
Samples						
PLA d0		67	143	148	2	373
PLA d2		58	146	153	22	362
PLA d4		66	135	154	58	371
PLA d7		-	138	154	64	369
PLA/CuHS d0		67	145	152	3	374
PLA/CuHS d2		57	147	152	8	356
PLA/CuHS d4		67	137	154	53	367
PLA/CuHS d7		60	136	154	66	267.5

**Table 4:** Main results obtained from the mechanical analysis of the samples tested. Data represent the mean values and standard deviation (*n* = 3). Identical letters mean no statistical significance, while different letters mean statistical significance (*p* < 0.05).

Samples	σ_max_ [MPa]	ε_max_ [%]	E [GPa]
PLA_d2	81 ± 13 ^a^	4 ± 1 ^A^	4.2 ± 0.5 ^aa^
PLA_d4	13 ± 5 ^b^	1.0 ± 0.3 ^A^	1.4 ± 0.2 ^ab^
PLA/CuHS_d2	27 ± 8 ^b^	7 ± 2 ^B^	1.3 ± 0.3 ^ab^
PLA/CuHS_d4	11 ± 3 ^b^	1.7 ± 0.2 ^A^	1.0 ± 0.1 ^ab^

**Table 5:** Characterization of the solid residue after the biodegradation process of PLA and PLA/CuHS 1% (*w*/*w*). Data represent the mean values and standard deviation (*n* = 4). Identical letters mean no statistical significance, while different letters mean statistical significance (*p* < 0.05).

Reactor	Dry mass_initial_ [%]	Dry mass_final_ [%]	R [%]	C/N_initial_	C/N_final_
Control	72 ± 3 ^a^	66 ± 3 ^b^	38 ± 2 ^A^	31 ± 2 ^aa^	36 ± 3 ^aa^
PLA	72 ± 3 ^a^	68 ± 3 ^b^	34 ± 3 ^A^	31 ± 2 ^aa^	30 ± 9 ^aa^
PLA/CuHS	72 ± 3 ^a^	67 ± 4 ^b^	35 ± 3 ^A^	31 ± 2 ^aa^	30 ± 5 ^aa^

## Data Availability

The original contributions presented in the study are included in the article. The raw data supporting the conclusions of this article will be made available by the authors on request.

## Supplementary Materials

The following supporting information can be downloaded at: https://www.mdpi.com/article/10.3390/polym16131772/s1, Figure S1: (a) Macroscopic aspect; (b) FESEM (scale 10 μm); (c) XRD with the assignment of the observed planes; and (d) Raman spectroscopy of the synthesized CuHS; Figure S2: Results corresponding to the thermal characterization of the composite films. (a) TGA; (b) DSC.; Figure S3: Comparison of Tg of PLA polymeric matrix without solvent and films made by solution casting using dichloromethane as solvent; Figure S4: pH monitoring through the composting process in the bioreactors; Figure S5: DSC thermograms of the first heating scan of the plastics residues at different degradations times; Table S1: Synthetic solid residue composition; Table S2: Microbial abundance in Log CFU/mL in the initial control suspensions and in the bacterial suspensions after 24 h of contact with the tested plastic samples.
