# Supplementary material for: Evaluation of PLA-Based Composite Films Filled with Cu2(OH)3NO3 Nanoparticles as an Active Material for the Food Industry: Biocidal Properties and Environmental Sustainability
Source: Polymers (Basel). 2024 Jun 23;16(13):1772. doi: 10.3390/polym16131772 (PMC11243838; doi:10.3390/polym16131772)
Supplement: Supplementary file 1 [file polymers-16-01772-s001.zip › polymers-3034951-supplementary.pdf]

polymers

MDPI

Supporting information

Evaluation of PLA-based composite films filled with $Cu_2(OH)_3NO_3$ nanoparticles as an active material for the food industry: biocidal properties and environmental sustainability

Xiomara Santos [1], Gabriela Domínguez [2], Juana Rodríguez [2], Javier Pozuelo [1], Manuel Hernández [2], Olga Martín [1,*], and Carmen Fajardo [2,*]

[1] Department of Materials Science and Engineering and Chemical Engineering, Higher Polytechnic School, Carlos III University of Madrid, Avenida Universidad 30, 28911 Leganés, Spain; xsantos@ing.uc3m.es (X.S.); jpozue@ing.uc3m.es (J.P.)
[2] Department of Biomedicine and Biotechnology, Faculty of Pharmacy, University of Alcalá, Ctra. Madrid-Barcelona km 33.6, 28805 Alcalá de Henares, Spain; juana.rodriguez@uah.es (J.R.); gabriela.dominguez@uah.es (G.D.); manuel.hernandez@uah.es (M.H.)
* Correspondence: martinc@ing.uc3m.es (O.M.); carmen.fajardo@uah.es (C.F.); Tel.: +34-91-885-46-76

Keywords: Compostability; polylactic acid; copper (II) hydroxynitrate; food packaging; bactericidal capacity; food microbiota.

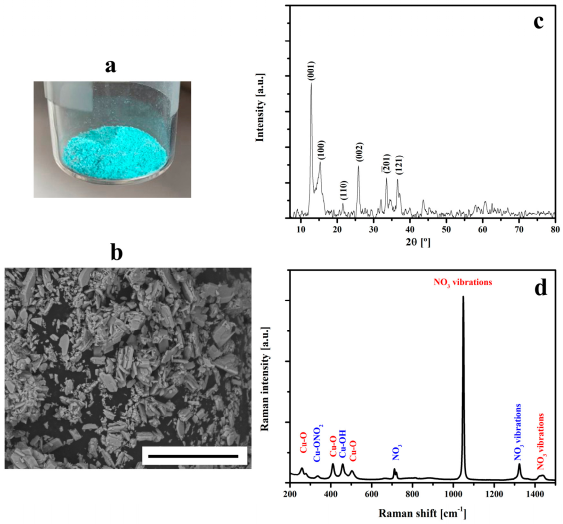

Figure S1. (a) Macroscopic aspect; (b) FESEM (scale 10 µm); (c) XRD with the assignment of the observed planes; and (d) Raman spectroscopy of the synthesized CuHS [1].

Table S1. Synthetic solid residue composition.

Material	Dry mass [%]
Sawdust	40
Rabbit food	30
Mature compost	10
Corn starch	10
Saccharose	5
Corn oil	4
Urea	1

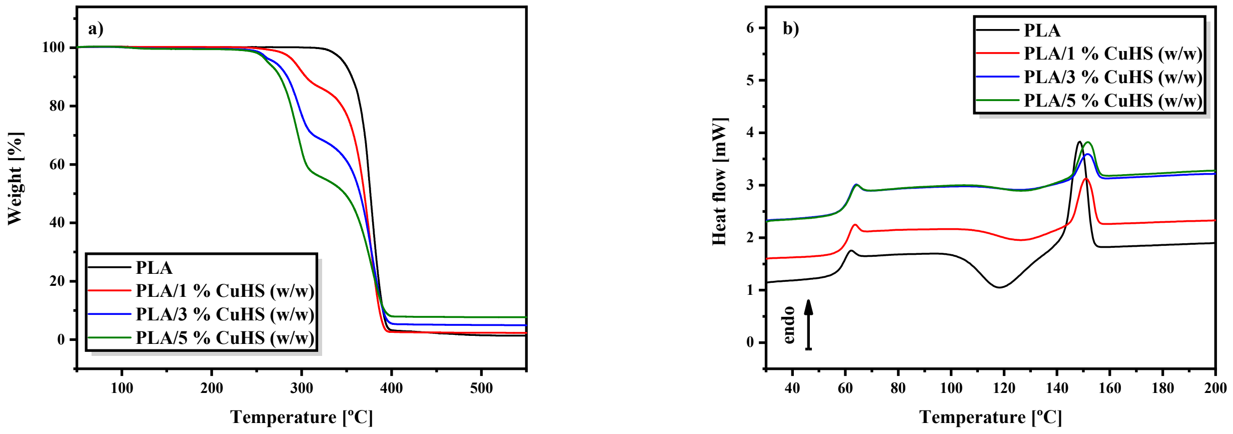

Figure S2. Results corresponding to the thermal characterization of the composite films. a) TGA; b) DSC.

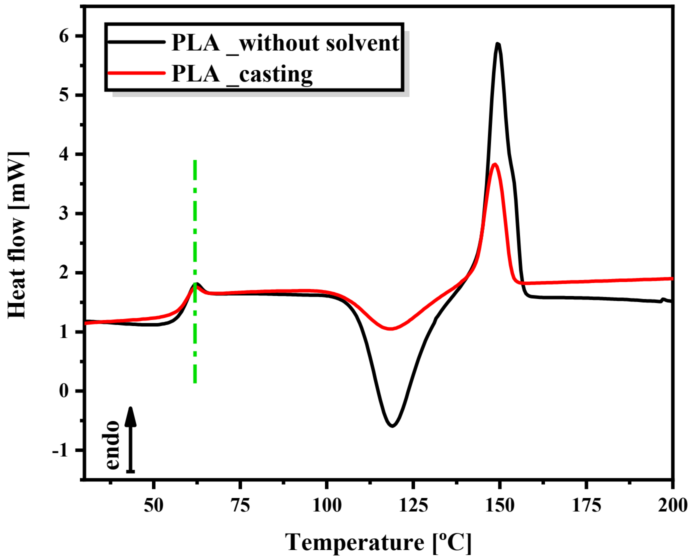

Figure S3. Comparison of Tg of PLA polymeric matrix without solvent and films made by solution casting using dichloromethane as solvent.

Table S2. Microbial abundance in Log CFU/mL in the initial control suspensions and in the bacterial suspensions after 24 hours of contact with the tested plastic samples.

Food	Samples	Microbial abundance (Log CFU/mL)
Chicken	Control	6.7 ± 0.1
	PLA	6.9 ± 0.1
	PLA/0.3 % CuHS (w/w)	6.7 ± 0.2
	PLA/1 % CuHS (w/w)	4.4 ± 0.6
	PLA/3 % CuHS (w/w)	nd
	PLA/5 % CuHS (w/w)	nd
Cheese	Control	5.7 ± 0.1
	PLA	6.5 ± 0.3
	PLA/0.3 % CuHS (w/w)	5.8 ± 0.3
	PLA/1 % CuHS (w/w)	1.2 ± 0.8
	PLA/3 % CuHS (w/w)	0.8 ± 0.8
	PLA/5 % CuHS (w/w)	nd
Fish	Control	6.0 ± 0.1
	PLA	7.3 ± 0.4
	PLA/0.3 % CuHS (w/w)	5.7 ± 0.8
	PLA/1 % CuHS (w/w)	nd
	PLA/3 % CuHS (w/w)	nd
	PLA/5 % CuHS (w/w)	nd

nd: no microbial growth detected

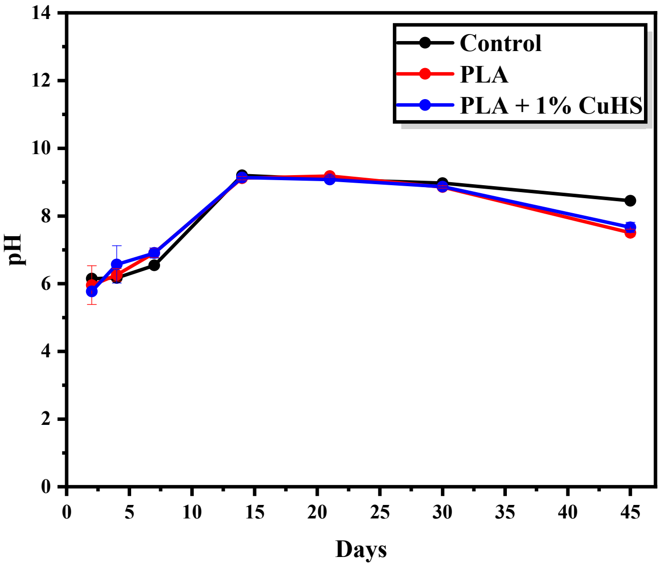

Figure S4. pH monitoring through the composting process in the bioreactors.

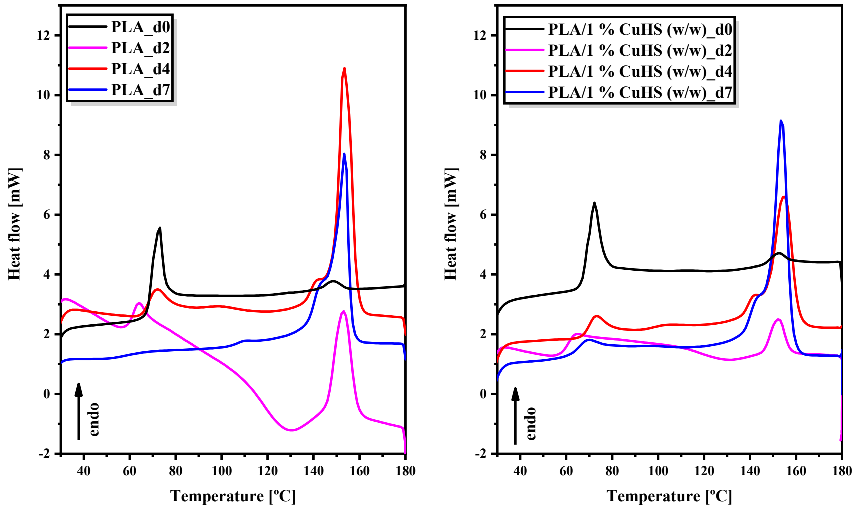

Figure S5. DSC thermograms of the first heating scan of the plastics residues at different degradations times.

References

1. Santos, X.; Rodríguez, J.; Guillén, F.; Pozuelo, J.; Molina-Guijarro, J.M.; Videira-Quintela, D.; Martín, O. Capability of Copper Hydroxy Nitrate ($Cu_2(OH)_3NO_3$) as an Additive to Develop Antibacterial Polymer Contact Surfaces: Potential for Food Packaging Applications. *Polymers* **2023**, 15, doi:10.3390/polym15071661.
